# Protein G: β‐galactosidase fusion protein for multi‐modal bioanalytical applications

**DOI:** 10.1002/btpr.3297

**Published:** 2022-08-29

**Authors:** Dana Motabar, Sally Wang, Chen‐Yu Tsao, Gregory F. Payne, William E. Bentley

**Affiliations:** ^1^ Fischell Department of Bioengineering University of Maryland College Park MD USA; ^2^ Institute for Bioscience and Biotechnology Research University of Maryland College Park MD USA; ^3^ Robert E. Fischell Institute for Biomedical Devices University of Maryland College Park MD USA

**Keywords:** beta‐galactosidase, electrochemical assays, protein engineering, protein G

## Abstract

β‐galactosidase (β‐gal) is one of the most prevalent markers of gene expression. Its activity can be monitored via optical and fluorescence microscopy, electrochemistry, and many other ways after slight modification using protein engineering. Here, we have constructed a chimeric version that incorporates a streptococcal protein G domain at the N‐terminus of β‐gal that binds immunoglobins, namely IgG. This protein G: β‐galactosidase fusion enables β‐gal‐based spectrophotometric and electrochemical measurements of IgG. Moreover, our results show linearity over an industrially relevant range. We demonstrate applicability with rapid spectroelectrochemical detection of IgG in several formats including using an electrochemical sensing interface that is rapidly assembled directly onto electrodes for incorporation into biohybrid devices. The fusion protein enables sensitive, linear, and rapid responses, and in our case, makes IgG measurements quite robust and simple, expanding the molecular diagnostics toolkit for biological measurement.

## 1 INTRODUCTION

In applications that span clinical and biotechnological or manufacturing settings, immunoglobin G (IgG) is an important bioanalytical component. Its level is a direct measure of physiological health in clinical settings, it is widely used as a recognition element in a variety of biotechnological applications (e.g., ELISA, histochemical, etc.) and therapeutic IgGs are a large and growing segment of the biopharmaceutical market. Protein G from *Streptococcus* is often used for IgG detection as it has high binding affinity to the Fc portion of both mouse and human IgG.^1^, ^2^ Correspondingly, protein G is often linked to a reporter enzyme which produces a response (e.g., colorimetric, luminescence, etc.) that can be correlated to IgG concentration. In our previous work,^3^ we showed that an electroassembled IgG capture interface comprised of thiolated polyethylene glycol (PEG) and a cysteinylated covalently coupled protein G, could be conveniently assembled directly onto a gold sensor interface. We then showed how commercially available protein G, when coupled with horseradish peroxidase (HRP), could provide an electrochemical readout of IgG titer when the interface was used to assess IgG titer in various bioprocessing samples. That said, the tetrameric enzyme β‐galactosidase (β‐gal)^4^, ^5^ expressed and purified from *Escherichia coli (E. coli)*, is also used in cellular and biomolecular applications and for decades has been a gold standard for localizing and assessing biomolecular function. β‐gal functions by hydrolyzing lactose and other β‐galactosides into monosaccharides.^5^ In many assays, this enzyme is used with the chromogenic substrate, ortho‐nitrophenyl‐β‐galactoside (ONPG), which is cleaved to produce a colorimetric response.^6^ Analogous substrates enable detection at various wavelengths and via fluorescence.^7^, ^8^, ^9^, ^10^, ^11^ Additionally, analogous to HRP, β‐gal can be used to produce an electrochemical response through the cleavage of 4‐Amino‐phenyl β‐D‐galactopyranoside (PAPG) yielding redox active p‐aminophenol (PAP).^12^, ^13^, ^14^, ^15^

We constructed a protein G: β‐gal fusion protein that can be used for analytical applications involving IgG. Importantly, because β‐gal exhibits activity toward a variety of substrates the fusion protein enables multiple modes of detection (i.e., optical, electrochemical). That is, addition of substrate, ONPG or PAPG, will produce either a spectrophotometric or electrochemical response, respectively. The fusion protein is easily expressed in *E. coli* and, owing to the inclusion of a hexahistidine tag at its N‐terminus, rapidly purified using immobilized metal affinity chromatography (IMAC). β‐gal provides a significant benefit compared to other reporter enzymes (e.g., horseradish peroxide, HRP) which involve more complicated electron transfer schemes as they often employ both a substrate and a redox mediator in order to enable an electrochemical response.^16^, ^17^ While β‐gal activity is associated with its tetrameric form,^18^, ^19^ chimeric proteins expressed from *E. coli* are free to associate into active conformations and importantly, since the protein when expressed in *E. coli* is not glycosylated, its use in evaluating an analyte's glycosylation state is facilitated.^3^ Overall, our protein G: β‐gal may serve as a versatile reporter in a variety of analytical applications.

## 2 MATERIALS AND METHODS

### 2.1 Construction and expression of protein G: β‐gal

The protein G: β‐gal, protein G only, and β‐gal only constructs were created using standard cloning methods (See Supplemental Note 1). Materials such as 4‐Amino‐phenyl β‐D‐galactopyranoside (PAPG), ortho‐Nitrophenyl‐β‐galactoside (ONPG), and human IgG from serum were purchased from Millipore Sigma (Burlington, MA). 4‐arm PEG thiol was purchased from JenKam (Plano, TX). 1,1′‐Ferrocenedimethanol (Fc) was purchased from Santa Cruz Biotechnology (Dallas, TX).

### 2.2 Bait prey assay

A bait‐pray assay was performed to evaluate protein G: β‐gal binding to IgG. About 730 μg of purified protein G: β‐gal (bait) was added to an Eppendorf tube (2 ml) containing 500 μl TALON metal affinity resin (Takara Bio; Mountain View, CA). About 500 μg of IgG (prey) was then added and allowed to incubate for 1 h at RT. After each respective incubation (protein G: β‐gal and IgG), three washes were performed for 5 min each using 0.1 M phosphate buffered saline (PBS), 0.05% Tween‐20. Bound proteins were eluted with elution buffer (20 mM phosphate, 0.5 M NaCl, 0.5 M imidazole, pH 7.4). Purified fractions were then run on an SDS‐PAGE gel for 45 min under 200 V. Experimental controls were also performed which included: (i) a “bait” only control in which the protein G: β‐gal was added to resin and eluted (no IgG was added) and, (ii) a “prey” only control in which the IgG is applied to the resin and eluted (no protein G: β‐gal was applied).

### 2.3 Surface plasmon resonance analysis

Human IgG was covalently bound to the surface of flow cell 2 of a CM5 chip to a final level of 1000 RU, using the NHS‐EDC kit from Cytiva Life Sciences and a BiaCore T200 instrument, also from Cytiva. Flow cell 1 was treated as a blank. Protein G, protein G: β‐gal, and protein G:HRP (0 to 500 nM in 120 μl of HBS‐EP buffer, 10 mM Hepes, 150 mM NaCl, 3 mM EDTA, 0.05% P20), were injected into flow cells 1 and 2 and binding was recorded for 2 min. The surfaces were washed with buffer for 3 min while the dissociation of analyte‐ligand complexes was followed time. The surfaces of the flow cells were regenerated by injecting 15 μl aliquots of 10 mM glycine, pH 1.5, and the process was repeated. For data analysis, sensorgrams were analyzed with BIAeval 3.1 software (Biacore). Values from the reference flow cell were subtracted to obtain the values for specific binding. Data were globally fitted to the Langmuir model of 1:1 binding.

### 2.4 Spectrophotometric and electrochemical characterization of β‐gal activity

For spectrophotometric detection, protein G: β‐gal (3.9 ‐125 μg/ml) was spiked with ONPG (1 mg/ml; Km value: 120 μM^14^, ^20^, ^21^) and the absorbance at 420 nm was measured every 15 s over a 5‐min interval. For electrochemical detection, PAPG (0.5 mg/ml; Km value: 18.7 ± 0.7 μM^22^) was added to the same concentrations of protein G: β‐gal. Cyclic voltammetry (CV) measurements were performed using a CHI6273c electrochemical analyzer (CH Instruments; Austin, TX) with a 3‐electrode system consisting of a standard 2 mM diameter gold disk electrode (working electrode; CH Instruments), a platinum wire (counter electrode) and an Ag/AgCl reference electrode. CVs were run from −0.1 to 0.4 V at a scan rate of 0.05 V/s every minute over a 10‐min interval. The peak oxidative current from the CVs was recorded and averaged. The lower limit of detection for the responses was calculated as described previously.^23^ To assess linearity, serially diluted IgG (0.16–5 μg/ml) was added to 96 well EIA/RIA microplates (Millipore Sigma) and incubated overnight at 4°C. Plates were washed three times (0.1 M PBS, 0.05% Tween‐20) and then blocked with bovine serum albumin (1%) for 1 h at RT. After washing three times, protein G: β‐gal was added (0.001, 0.01, and 0.1 g/L) and incubated for 1 h at RT. Wells were subsequently washed three times. For spectrophotometric responses, ONPG (1 mg/ml) was added to the wells and allowed to incubate for various times depending on protein G: β‐gal concentration (4, 6, and 12 min). To stop the reactions, NaHCO_3_ (1 M) was added and absorbance measurements at 420 nm were taken.

### 2.5 Evaluation using a hydrogel detection interface

A thiolated polyethylene glycol hydrogel interface was electroassembled as described previously.^3^ Briefly, the interfaces were constructed by immersing the surface of a 2 mm diameter gold standard electrode (working electrode) in a solution containing Fc (5 mM) and PEG thiol (50 mg/ml) along with a platinum wire (counter electrode) and an Ag/AgCl reference electrode. PEG electrodeposition occurred for 1 minute at a constant potential of 0.4 V. After PEG hydrogel formation, remaining sulfhydryl groups were oxidized by immersing the surface in a solution of Fc (5 mM) and applying a constant voltage of 0.4 V for 2 min. For the titer detection interface, the PEG‐coated electrodes were immersed in cysteinylated protein G (250 μg/ml in 0.1 M PBS, pH 7.4) overnight. After 3 rinses with wash buffer (0.1 M PBS, 0.05% Tween‐20, pH 7.4), the interfaces were sequentially incubated in serially diluted concentrations of IgG (0–1000 μg/ml) and protein G: β‐gal (0.01 g/L), respectively, for 1 h at room temperature. After each respective incubation (IgG and protein G: β‐gal), 3 washes were performed for 5 min each with 0.1 M PBS, 0.05% Tween‐20. Current measurements were then performed using constant potential amperometry. Specifically, electrodes were submerged in a stirred beaker containing 0.1 M potassium phosphate buffer under an applied potential of 0.4 V. PAPG (0.5 mg/ml) was spiked into to the mixtures and the increase in current from the baseline was recorded. For spectrophotometric measurements, electrodes were incubated in Eppendorf tubes containing ONPG (1 mg/ml) and incubated at 37°C on a shaker plate (shaking at approximately 100 RPM) for 5 min and then absorbance measurements at 420 nm were taken. The lower limit of detection for the responses was calculated as described previously.^23^

## 3 RESULTS AND DISCUSSION

### 3.1 Binding protein G: β‐gal to IgG

**FIGURE 1:**
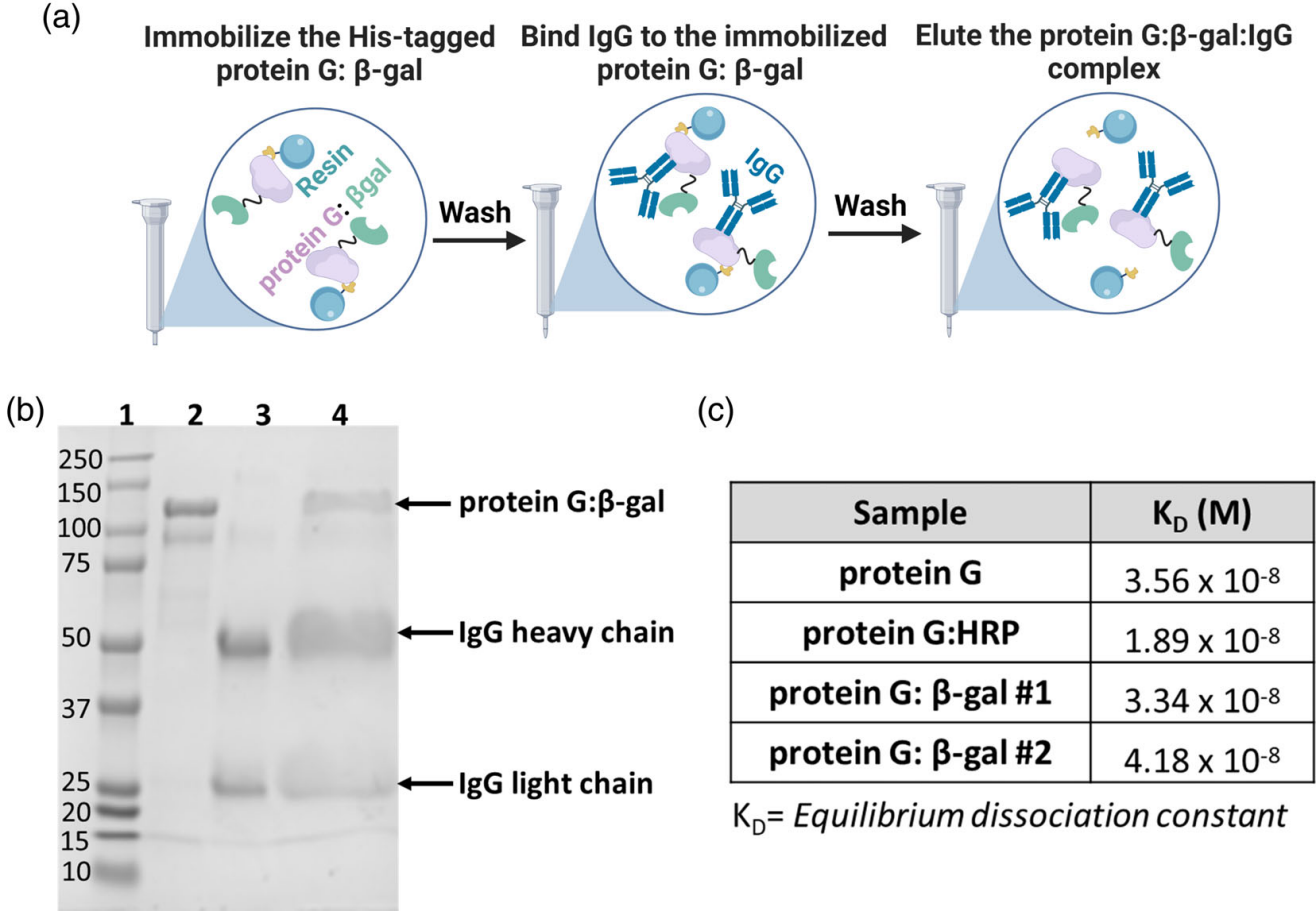
(a) Bait‐prey assay experimental protocol. (b) SDS‐PAGE results of bait‐prey assay: ladder (lane 1), protein G:β‐gal (lane 2), IgG (lane 3), and bait‐prey assay eluate (lane 4). All lanes loaded with 10 μg protein. The presence of protein G: β‐gal and IgG in the eluate indicate that protein G: β‐gal binds IgG. (c) SPR experiments indicate that the IgG binding capacity for protein G, protein G: HRP, and protein G: β‐gal (two separate preparations) is consistent

As illustrated in Figure 1a, a bait‐prey assay was performed. The His‐tagged protein G:β‐gal expressed and purified from *E. coli* (see Supplemental Note 1) bound the IMAC resin and served as “bait.” IgG, serving as “prey,” was next added to the mixture and bound to protein G. The “bait‐prey” complex was then eluted from the column using imidazole and the fractions were analyzed via SDS‐PAGE (Figure 1b). The band corresponding to protein G: β‐gal (~126 kDa, lane 2), the IgG heaving chain (~50 kDa, lane 3), and the IgG light chain (~25 kDa, lane 3) all aligned nicely with the molecular weight ladder (lane 1). Also, the IMAC eluate (lane 4) clearly showed three distinct bands aligning with the molecular weights of protein G: β‐gal, IgG heavy chain, and IgG light chain, respectively. Because the eluate contained both protein G: β‐gal and IgG, these data confirm that IgG was bound to the protein G: β‐gal that, in turn, was immobilized on the IMAC resin. Additional supportive data are shown in Figure S2. We also wanted to quantitatively compare the IgG binding capability of protein G: β‐gal to both a protein G only control and the similar commercially available reporter (protein G:HRP). For this analysis, protein G: β‐gal from two different sample preparations (protein expression and purification) were used in order to assess batch‐to‐batch reproducibility. The samples were then evaluated using surface plasmon resonance (SPR) and the results from the SPR kinetic analyses are shown in Figure 1c and Figure S2B. For protein G, protein G: HRP, and both preparations of protein G: β‐gal, we observed strong binding to IgG with all samples having similar equilibrium dissociation constants (*K*_D_ = 3.56E × 10^−8^, 1.89 × 10^−8^, and 3.34 × 10^−8^/4.18 × 10^−8^, respectively). In sum, these results demonstrate that protein G: β‐gal binding affinity, when expressed and purified from *E. coli*, is similar to the commercially available protein G:HRP as well as protein G without a fusion partner.

### 3.2 Spectrophotometric and electrochemical responses

**FIGURE 2:**
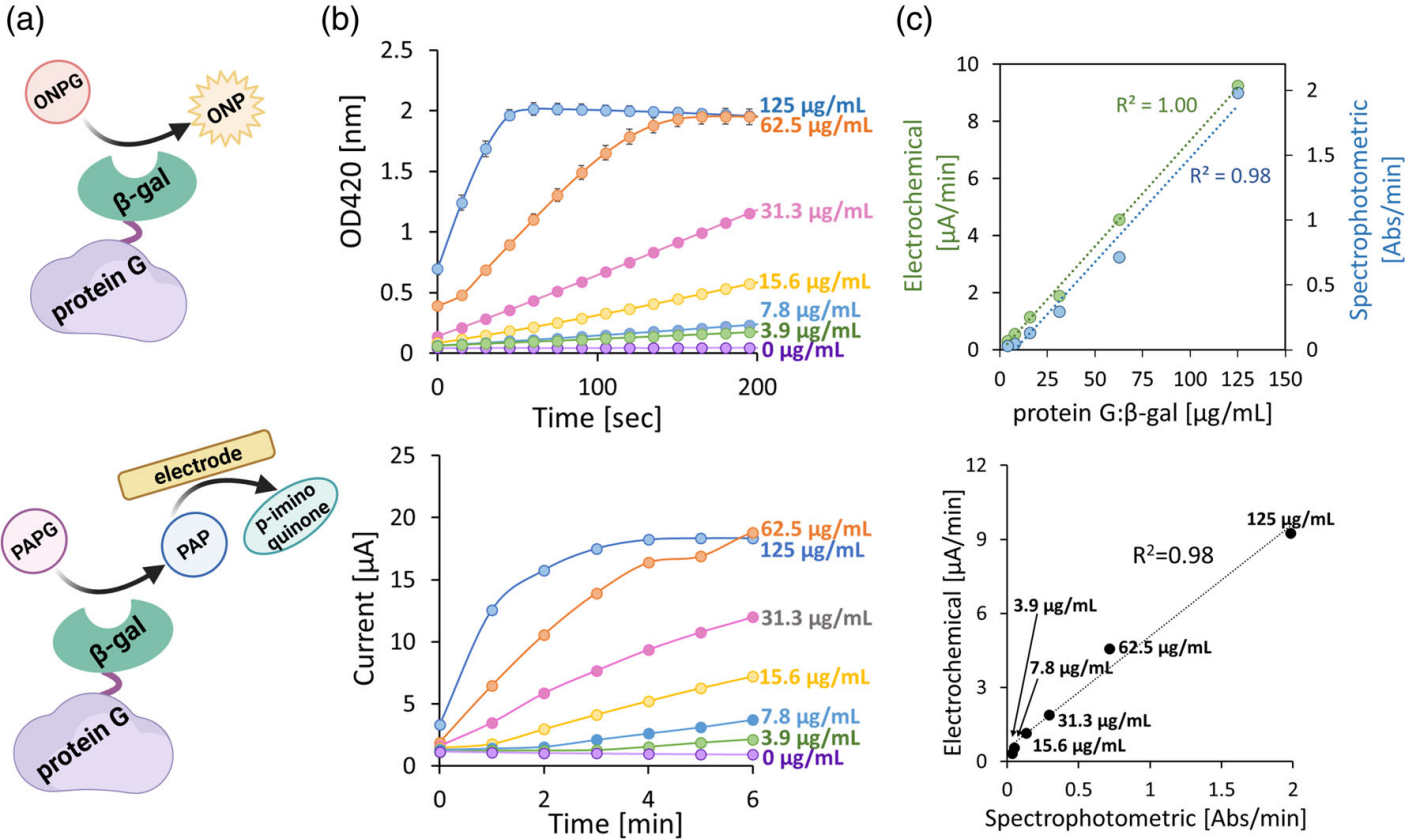
(a) Protein G:β‐gal produces spectrophotometric (top) and electrochemical (bottom) responses using the substrates ONPG and PAPG, respectively. (b) Spectrophotometric (top) and electrochemical (bottom) responses increased as a function of protein G:β‐gal concentration (3.9–125 μg/ml). (c) Linear increase in electrochemical and spectrophotometric responses as a function of protein G: β‐gal concentration. Observed a linear correlation (*R* ^2^ = 0.98) between the spectrophotometric and electrochemical responses (bottom). Values are based on the initial rates shown in Figure 2b

We next wanted to determine protein G: β‐gal functionality in both electrochemical and spectrophotometric settings. In Figure 2a, ONPG is cleaved to produce o‐nitrophenyl (ONP), which generates a yellow color that is measured by absorbance at 420 nm. For our measurements, serially diluted samples of protein G: β‐gal (3.9–125 μg/ml) were spiked with 4 mg/ml ONPG and the absorbance was measured every 15 s over a 5‐minute interval. Correspondingly, PAPG was also cleaved by β‐gal but produced p‐aminophenol (PAP) (lower scheme of Figure 2a), which can be electrochemically detected by oxidation to *p*‐iminoquinone at the electrode (see Methods). Here, a bare gold electrode was incubated with serially diluted samples of protein G: β‐gal (3.9–125 μg/m). Next, PAPG was added to the solution, electrochemical cyclic voltammetry (CV) measurements were taken every minute over a 10‐min interval, and the peak oxidative currents from the CV measurements (Figure S3A) were recorded. As expected, for both detection schemes, higher concentrations of protein G: β‐gal produced stronger and more rapid responses (Figure 2b). The data depicted illustrate the rapid responses that are dependent on protein quantity. The data in Figure 2C (top) demonstrate linearity for each. Both the electrochemical (*R* ^2^ = 1.00) and spectrophotometric (*R* ^2^ = 0.98) assays were linear over the full range of protein G:β‐gal tested (3.9–125 mg/ml). The lower limit of detection for the methods was calculated to be 10.8 μg/ml (spectrophotometric) and 19.8 μg/ml (electrochemical). In the lower panel of Figure 2c, we plot the electrochemical measurements as a function of the spectrophotometric method. Naturally, the calculated rates for both modalities were linear relative to each another across the entire concentration range evaluated (*R* ^2^ = 0.98). The spectrophotometric and electrochemical responses of β‐gal only (enzyme only) were performed to confirm that the fusion protein configuration had no effect upon the responsiveness of the enzyme. For both measurement modalities, the results in Figure S3B show that there is minimal difference between β‐gal and protein G: β‐gal. Lastly, the storage stability of the fusion protein was characterized by performing freeze thaw studies. Results are shown in Figure S3C. Protein G: β‐gal maintained its spectrophotometric and electrochemical reaction rates (shown in the table) for two freeze thaw cycles (reaction rates within 10% variance of initial response).

### 3.3 Spectrophotometric measurement of IgG

**FIGURE 3:**
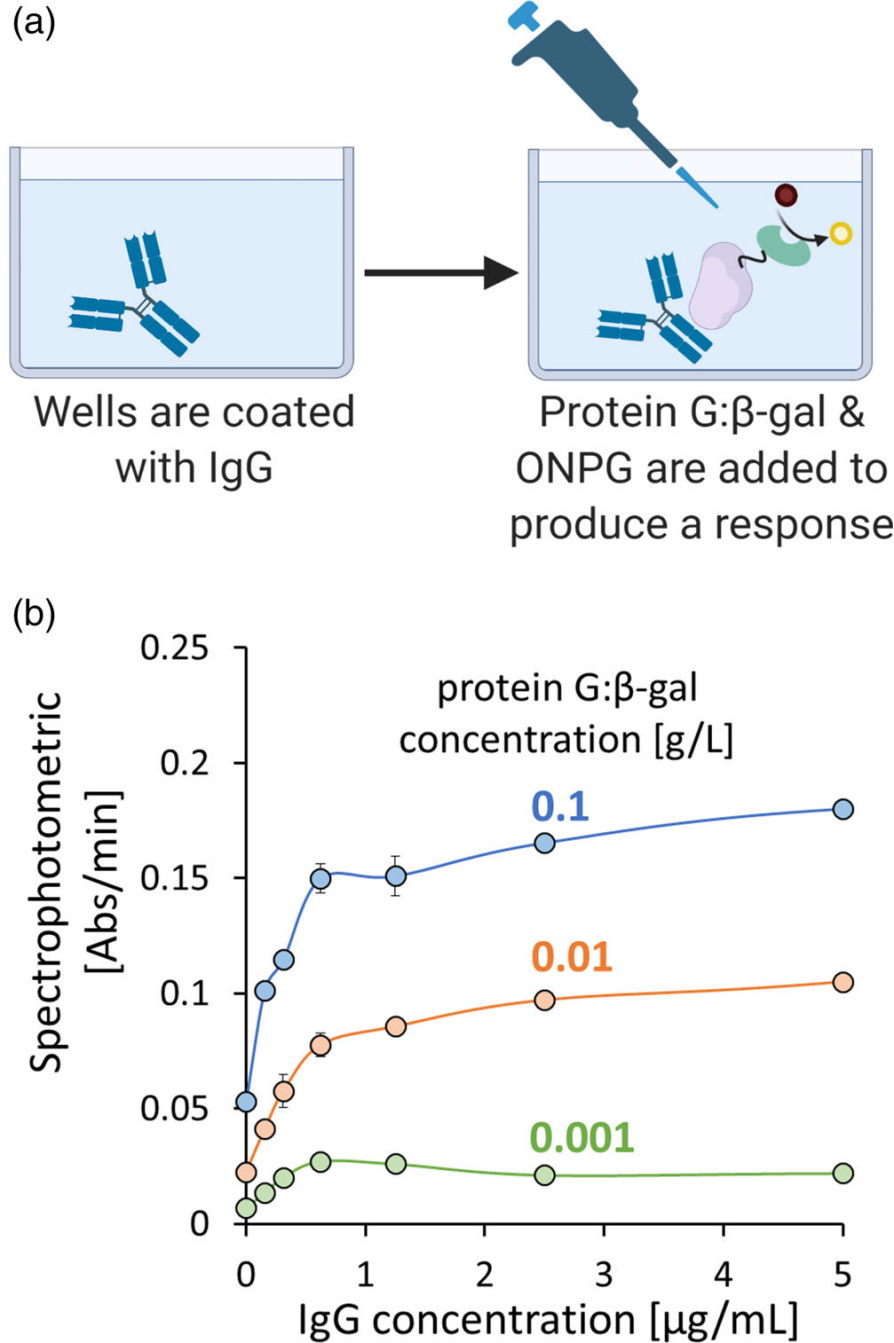
(a) Experimental protocol to evaluate the concentration‐dependent response of protein G:β‐gal. (b) Spectrophotometric response increased as a function of protein G:β‐gal concentration (*n* = 3)

We next wanted to evaluate function in fixed formats. In Figure 3a, a microplate with high protein binding wells was incubated overnight with serially diluted IgG (0.16–5 μg/ml). After washing to remove nonspecific binding, protein G: β‐gal (0.001–0.1 g/L) was added and allowed to incubate for 1 h. ONPG was then added and absorbance (l = 420 nm) measurements were taken (Figure 3b). As expected, both increased IgG and protein G:β‐gal concentrations resulted in higher responses. Our results, which show saturation at IgG concentrations above 1 mg/ml, suggest that there was significant and perhaps excess binding capacity for additional protein G: β‐gal. We note that IgG has two protein G binding sites on its Fc region. This allows for increased binding per IgG and perhaps these additional sites become increasingly occupied with increased protein G: β‐gal, akin to positive cooperativity. The saturation phenomena was confirmed by replotting the data in a Lineweaver‐Burke type plot (Figure S4).

### 3.4 Evaluation using a hydrogel detection interface

**FIGURE 4:**
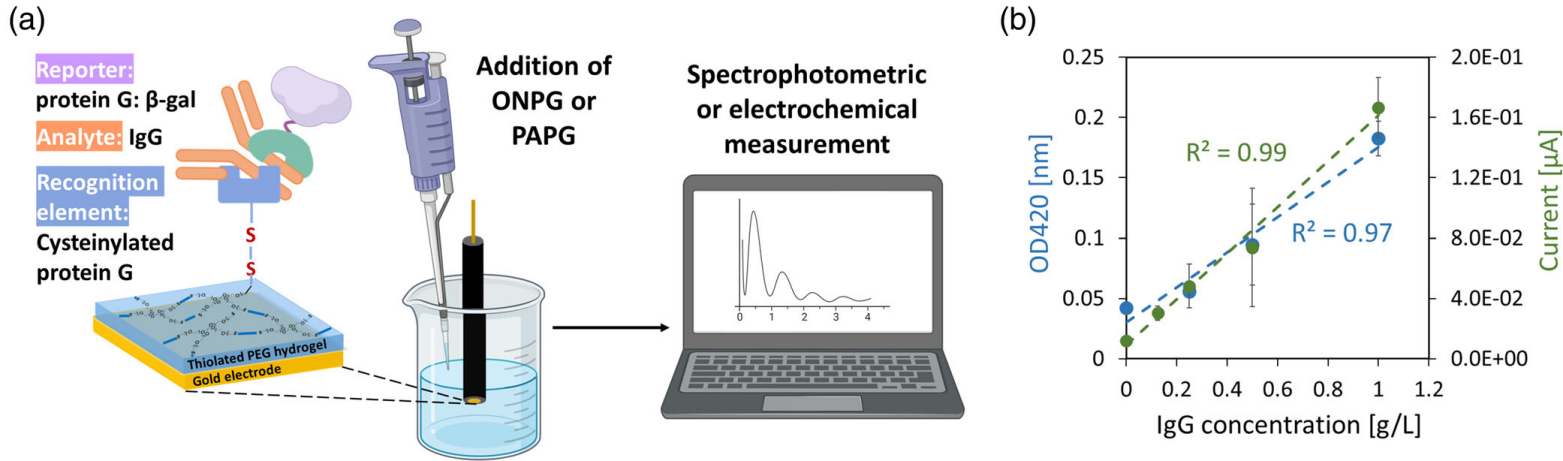
(a) Sensor interface with protein G:β‐gal as the reporter produces spectrophotometric and electrochemical outputs that can be correlated to the concentration of IgG in a sample. (b) Observed linear spectrophotometric (*R* ^2^ = 0.97) and electrochemical (*R* ^2^ = 0.99) responses across IgG concentrations (*n* = 3)

Lastly, we wanted to demonstrate the capability of protein G: β‐gal when used in an electroassembled sensor format. We first created a sensor interface directly assembled onto sensing electrodes via electronic signals, as described above,^3^ and then used the same electrode to assess IgG level in a complex sample. This electroassembly methodology enables spatially defined functionalization of electronic surfaces.^24^, ^25^ Figure 4a illustrates the configuration and use of the interface. Here, an initial layer of a thiolated polyethylene glycol (PEG) is electrodeposited as a cross‐linked hydrogel onto the surface of a gold electrode by applying a charge in the presence of a redox mediator (ferrocene).^26^ While the previous efforts have assembled polysaccharides such as chitosan,^27^, ^28^, ^29^ the use of thiolated PEG here minimizes non‐specific binding of various contaminants to an electrode‐based sensor.^26^, ^30^ The PEG is crosslinked by the oxidative coupling of PEG thiols into disulfide bonds. The electroassembled hydrogel is then overlayed with the recognition element (cys‐tagged protein G^31^) whose cysteine tag engineered onto its C‐terminus allows it to covalently bond via disulfide bonds under an additional oxidative charge to the electrode (some thiol groups of the PEG are electrochemically oxidized to sulfenic acid and this covalently couples to the thiols of the terminal cysteines engineered onto protein G). This hydrogel film thus forms the detection interface.^3^, ^26^, ^31^ To measure IgG, the detection interface was then incubated with IgG (serving as the analyte) and protein G: β‐gal of this work (serving as the reporter). Shown in Figure S5A, circular interfaces were constructed, functionalized with protein G and IgG and then FITC‐labeled protein G:β‐gal was allowed to bind. The near uniform green color suggests near uniform binding (see Methods). Then, with constructed interfaces and using identical incubations in IgG (0–1000 μg/ml; 0–6.7 μM) and protein G: β‐gal (0.01 g/L; 0.08 μM), both spectrophotometric and electrochemical responses to ONPG and PAPG additions were taken. The transient electronic currents produced from the interfaces by adding PAPG into the mixture are depicted. The responses were rapid, reaching maxima within seconds (Figure S5B). For spectrophotometric evaluation, the interfaces were incubated in ONPG for 5 min at 37°C and the absorbances were measured. The averaged peak currents and absorbance measurements at 5 min, respectively, were then depicted in Figure 4b. For both modalities, the results indicate that the electrochemical (*R* ^2^ = 0.99) and spectrophotometric (*R* ^2^ = 0.97) responses were robust and linear over a large concentration range of IgG (0–1000 μg/ml). The lower limit of detection was calculated to be 44 μg/ml (electrochemical) and 127 μg/ml (spectrophotometric). This range of detection is significant for therapeutic IgGs which can have wide titer range depending on the product^32^, ^33^; measurement ranges for IgG can be optimized by performing appropriate dilutions of IgG and protein G: β‐gal to ensure that the response is within the linear range of the assay. Importantly, these results also confirm that *both* spectrophotometric and electrochemical modalities can be facilitated within the same electrode assembled assay system, providing both redundancy and independent confirmation. Controls without cysteinylated protein G, protein G: β‐gal, or IgG are shown in Figure S5C for both the spectrophotometric (left) and electrochemical (right) modalities. In sum, our results confirm that the protein G: β‐gal fusion protein can be used to accurately enable quantification of IgG in a variety of settings.

## 4 CONCLUSIONS

We developed a protein G: β‐gal fusion protein that enables both solution‐based and electrode‐based spectrophotometric and electrochemical assessment of IgG. Its substitution for protein G: HRP simplifies the electrochemical assessment of IgG titer described in our previous report and owing to the ease with which it can be assembled onto electrodes, might enable simultaneous measurement of glycan structure and IgG titer. Naturally, further evaluation with complex samples (IgG of different subclasses, IgG in cell culture media, IgG in process storage vessels, etc.) is needed to better understand the effectiveness of the fusion protein. Moreover, it may prove interesting to determine if PAPG and ONPG can be used together for simultaneous spectrophotometric and electrochemical measurement, as added redundancy. We suggest that the new protein G: β‐gal will expand upon the analytical toolkit by providing sensitive, linear, and rapid multi‐modal responses.

## AUTHOR CONTRIBUTIONS

**Dana Motabar:** Conceptualization (equal); data curation (equal); formal analysis (equal); methodology (equal); writing – original draft (equal). **Sally Wang:** Data curation (equal); investigation (equal); methodology (equal). **Chen‐Yu Tsao:** Conceptualization (equal); data curation (equal); formal analysis (equal); investigation (equal); methodology (equal). **Gregory F. Payne:** Conceptualization (equal); project administration (equal); supervision (equal); writing – review and editing (equal). **William Bentley:** Conceptualization (equal); funding acquisition (equal); supervision (equal); visualization (equal); writing – original draft (equal); writing – review and editing (equal).

## Supporting information


**Appendix S1** Supporting Information

## Data Availability

All data are available upon request of the authors.
