# Supplementary material for: Protein G: β‐galactosidase fusion protein for multi‐modal bioanalytical applications
Source: Biotechnol Prog. 2022 Aug 29;38(6):e3297. doi: 10.1002/btpr.3297 (PMC10078426; doi:10.1002/btpr.3297)
Supplement: Supplementary file 1 — Appendix S1 Supporting Information [file BTPR-38-0-s001.docx]

Supplementary Material

**Protein G: β-galactosidase fusion protein for multi-modal bioanalytical applications**

Dana Motabar^1,2,3^, Sally Wang^1,2,3^, Chen-Yu Tsao^1,2,3^, Gregory F. Payne^2,3^, William E. Bentley^1,2,3^

^1^Fischell Department of Bioengineering, University of Maryland, College Park, Maryland 20742, United States

^2^Institute for Bioscience and Biotechnology Research, University of Maryland, College Park, Maryland 20742, United States

^3^Robert E. Fischell Institute for Biomedical Devices, University of Maryland, College Park, Maryland 20742 United States

**Correspondence:**

William E. Bentley

Fischell Department of Bioengineering,

Institute for Bioscience and Biotechnology Research,

Robert E. Fischell Institute for Biomedical Devices,

University of Maryland, College Park, Maryland, 20742, United States

Email: [bentley@umd.edu](mailto:bentley@umd.edu)

**Supplemental Note 1 – Plasmid Assembly, Expression, and Purification**

Briefly, the Xpress tag of pET200-D-lacZ (Thermo Fisher Scientific; Waltham, MA) was removed. The vector and gene for protein G were then digested with BamHI-HF and BsrGI-HF restriction enzymes. The gene for protein G was then ligated into the modified pET200-D-lacZ vector (also serving as the β-gal only control) between the N-terminal His_6_ tag and the C-terminal *lacZ* gene. The resultant plasmid map is illustrated in **Figure S1A** and the amino acid sequence is shown below. For the protein G only control, the *lacZ* gene was deleted from the construct. The plasmids were transformed into BL21 Star™ (DE3) One Shot® *E. coli* for protein expression. The fusion protein and the respective controls (protein G only and β-gal only) were overexpressed by 1 mM IPTG induction when cell densities reached OD_600_ = 0.4 at 37 °C. Cells grown overnight were harvested at room temperature (RT) by centrifugation (14000 x g) at 4 °C for 20 min. Cells were lysed by incubation in BugBuster solution (Novagen; Madison, WI) at RT for 40 minutes, the soluble cell extracts were additionally sonicated for 10 minutes and then centrifuged to remove cell debris. The cell extracts were then loaded onto 5mL HiTrap columns (GE Healthcare; Chicago, IL) and the bound target proteins were washed with binding buffer (20 mM phosphate, 0.5 M NaCl, 40 mM imidazole, pH 7.4) to remove non-specifically bound proteins. The fusion protein was eluted from the column (20 mM phosphate, 0.5 M NaCl, 0.5 M imidazole, pH 7.4). The purified proteins were then buffer exchanged using Slide-A-Lyzer dialysis cassettes (Thermo Fisher Scientific; Waltham, MA) into 0.1 M phosphate buffered saline, pH 7.4 for further experiments. The yield of the purified fusion protein was calculated to be 34.5 μg per mL of IPTG-induced BL21 (DE3) culture (OD600 = 1.1). SDS-PAGE of the soluble fraction was run and the Coomassie blue stained gel (**Figure S1B**) shows that protein G: β-gal is highly expressed in the culture.


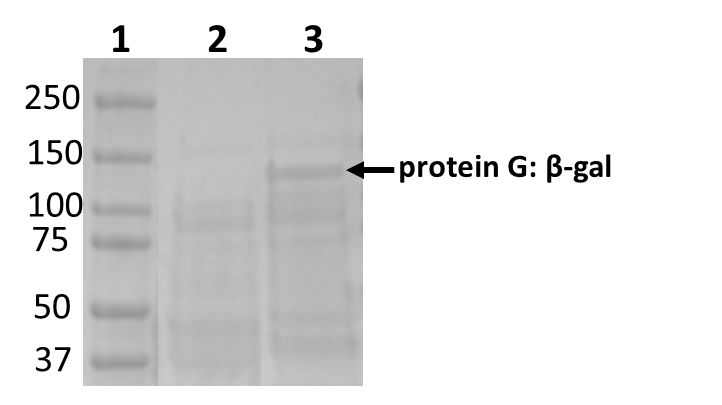


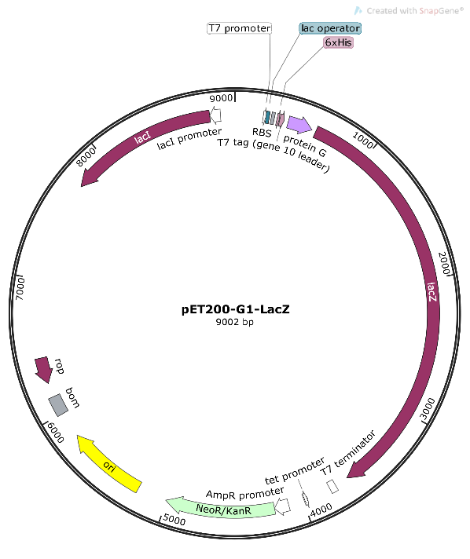


(A)

(B)

**Figure S1.** (A) Plasmid map of the protein G: β-gal construct. (B) SDS-PAGE shows that protein G: β-gal is the predominantly expressed protein in the cell culture. Lane 1 contains the ladder. Lane 2 contains the soluble fraction of IPTG-induced BL21 (DE3) culture without the plasmid (pET-200-G1-LacZ). Lane 3 contains the contains the soluble fraction of IPTG-induced BL21 (DE3) culture with the plasmid (pET-200-G1-LacZ).

**Amino acid sequence of protein G:β-gal:**

HHHHHHGMASMTGGQQMGREDPMGVKIRMTTYKLVINGKTLKGETTTKTVDAETAEKAFKQYANDNGVDGVWTYDDATKTFTVTEMDPNVHHPFTIDPVVLQRRDWENPGVTQLNRLAAHPPFASWRNSEEARTDRPSQQLRSLNGEWRFAWFPVPEAVPESWLECDLPEADTVVVPSNWQMHGYDAPIYTNVTYPITVNPPFVPTENPTGCYLTFNVDESWLQEGQTRIIFDGVNSAFHLWCNGRWVGYGQDSRLPSEFDLSAFLRAGENRLAVMVLRWSDGSYLEDQDMWRMSGIFRDVSLLHKPTTQISDFHVATRFNDDFSRAVLEAEVQMCGELRDYLRVTVSLWQGETQVASGTAPFGGEIIDERGGYADRVTLRLNVENPKLWSAEIPNLYRAVVELHTADGTLIEAEACDVGFREVRIENGLLLLNGKPLLIRGVNRHEHHPLHGQVMDEQTMVQDILLMKQNNFNAVRCSHYPNHPLWYTLCDRYGLYVVDEANIETHGMVPMNRLTDDPRWLPAMSERVTRMVQRDRNHPSVIIWSLGNESGHGANHDALYRWIKSVDPSRPVQYEGGGADTTATDIICPMYARVDEDQPFPAVPKWSIKKWLSLPGETRPLILCEYAHAMGNSLGGFAKYWQAFRQYPRLQGGFVWDWVDQSLIKYDENGNPWSAYGGDFGDTPNDRQFCMNGLVFADRTPHPALTEAKHQQQFFQFRLSGQTIEVTSEYLFRHSDNELLHWMVALDGKPLASGEVPLDVAPQGKQLIELPELPQPESAGQLWLTVRVVQPNATAWSEAGHISAWQQWRLAENLSVTLPAASHAIPHLTTSEMDFCIELGNKRWQFNRQSGFLSQMWIGDKKQLLTPLRDQFTRAPLDNDIGVSEATRIDPNAWVERWKAAGHYQAEAALLQCTADTLADAVLITTAHAWQHQGKTLFISRKTYRIDGSGQMAITVDVEVASDTPHPARIGLNCQLAQVAERVNWLGLGPQENYPDRLTAACFDRWDLPLSDMYTPYVFPSENGLRCGTRELNYGPHQWRGDFQFNISRYSQQQLMETSHRHLLHAEEGTWLNIDGFHMGIGGDDSWSPSVSAEFQLSAGRYHYQLVWCQKAAARV*


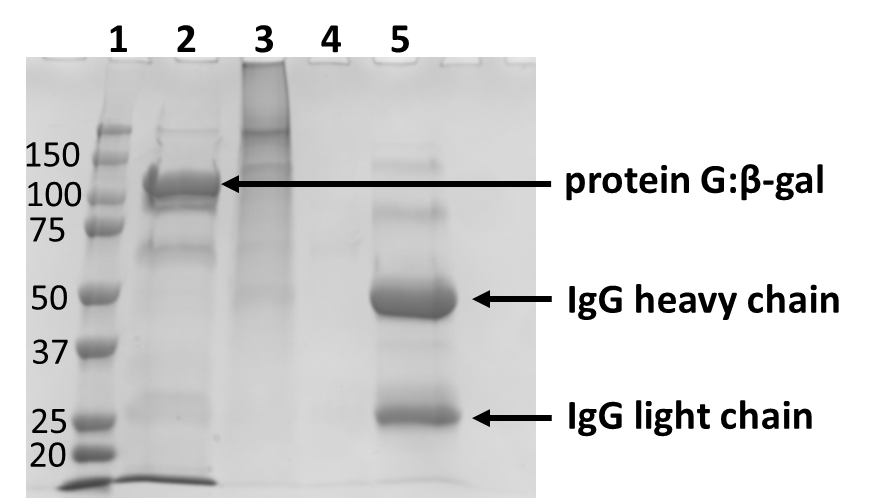


(A)


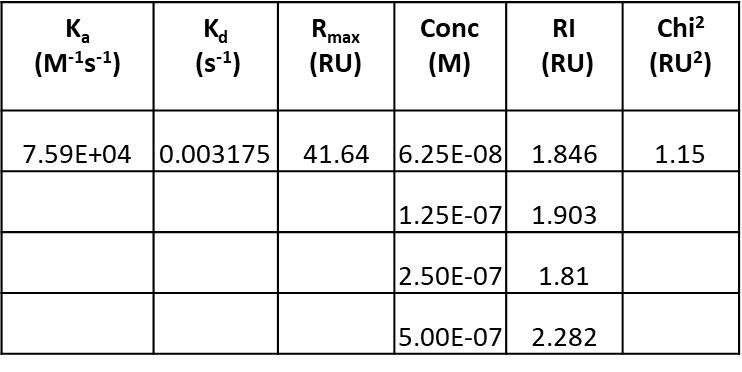

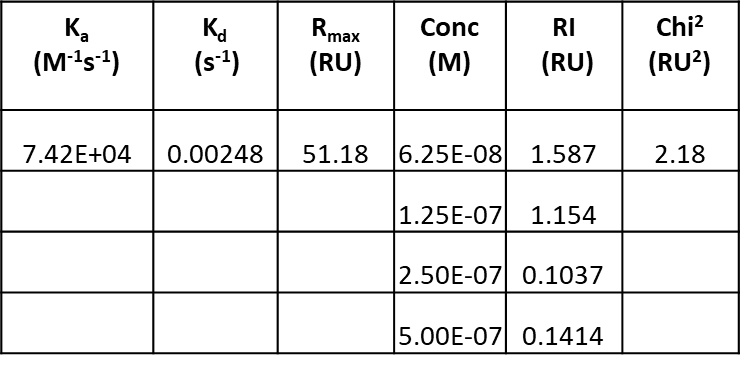

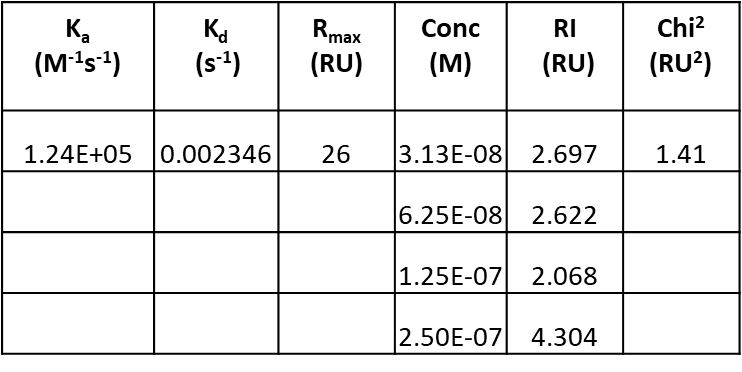

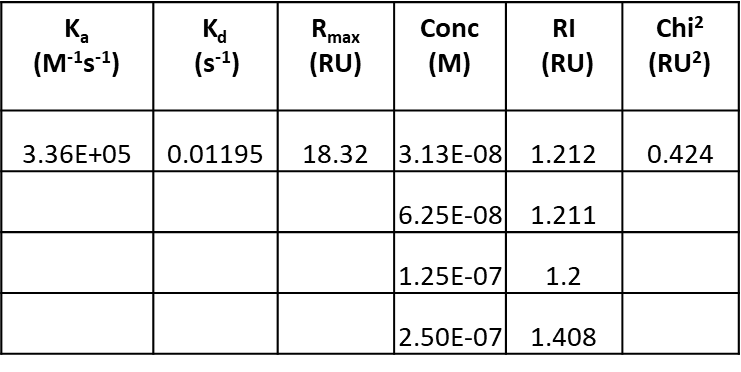


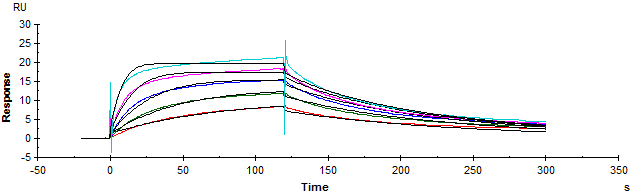


**Protein G**

(B)

**Figure S2.** (A) SDS-PAGE of the controls from the bait prey assay. Lane 1 contains the ladder. Lane 2 and 4 contain the fractions from the “bait” only control. For the “bait” only control, the protein G: β-gal was added to resin and eluted (no IgG was added).


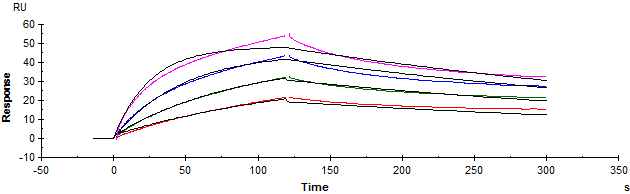


**Protein G: β-gal #1**

k_a_=*Association rate constant*

k_d_=*Dissociation rate constant*


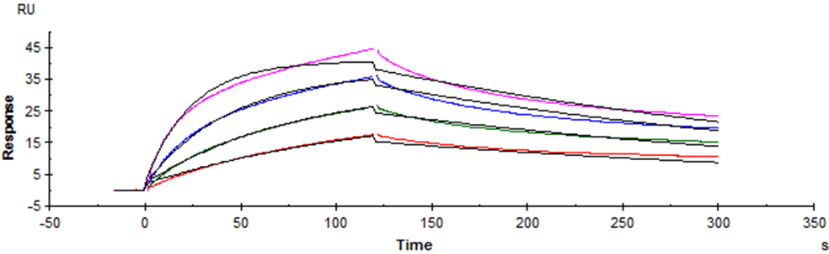


**Protein G: β-gal #2**


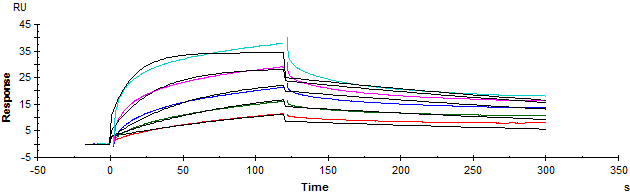


**Protein G: HRP**

As expected, a single band corresponding to protein G: β-gal was evident in the elution fraction (lane 2) and no protein G: β-gal band was visible in the flow through fraction (lane 4), indicating that the His-tagged protein bound the IMAC resin. Lane 3 and 5 contain the fractions from the “prey” only control. For the “prey” only control, the IgG is applied to the resin and eluted (no protein G: β-gal was applied). The “prey” only elute (lane 3) and its corresponding flow through fraction (lane 5) showed that IgG does not bind to the resin, confirming that the IgG is only binding to protein G: β-gal. All lanes loaded with 10 μg protein. (B) Representative sensorgrams and corresponding SPR binding analysis of protein G, protein G:HRP, and protein G:β-gal (from two separate sample preparations) to human IgG.


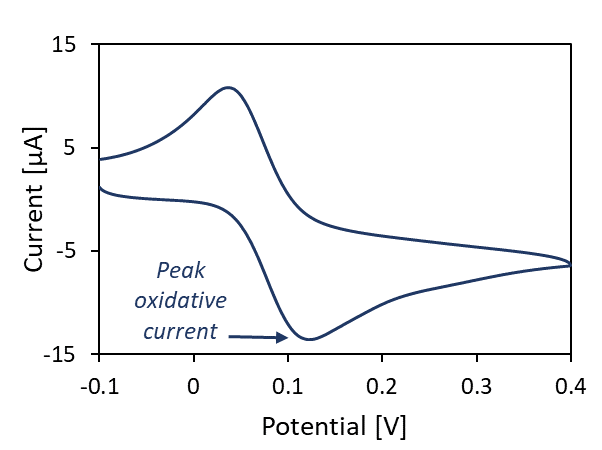


(C)

(B)

(A)


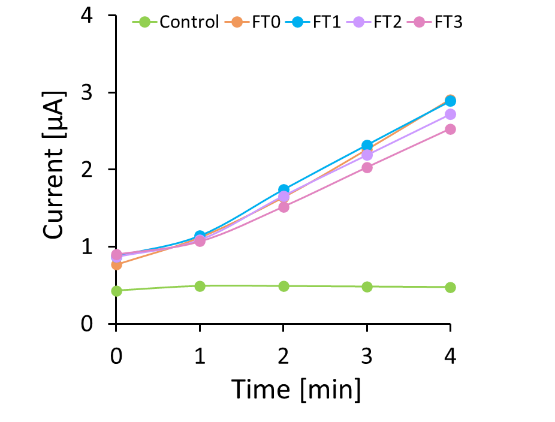

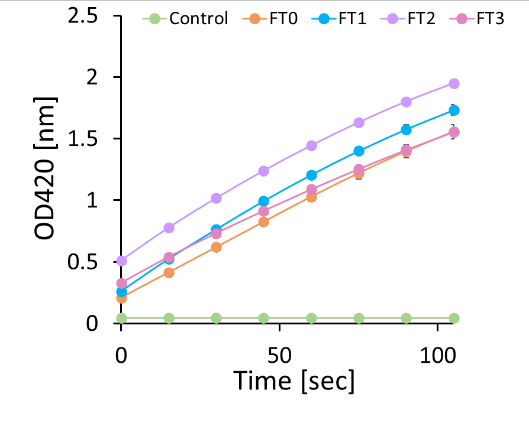

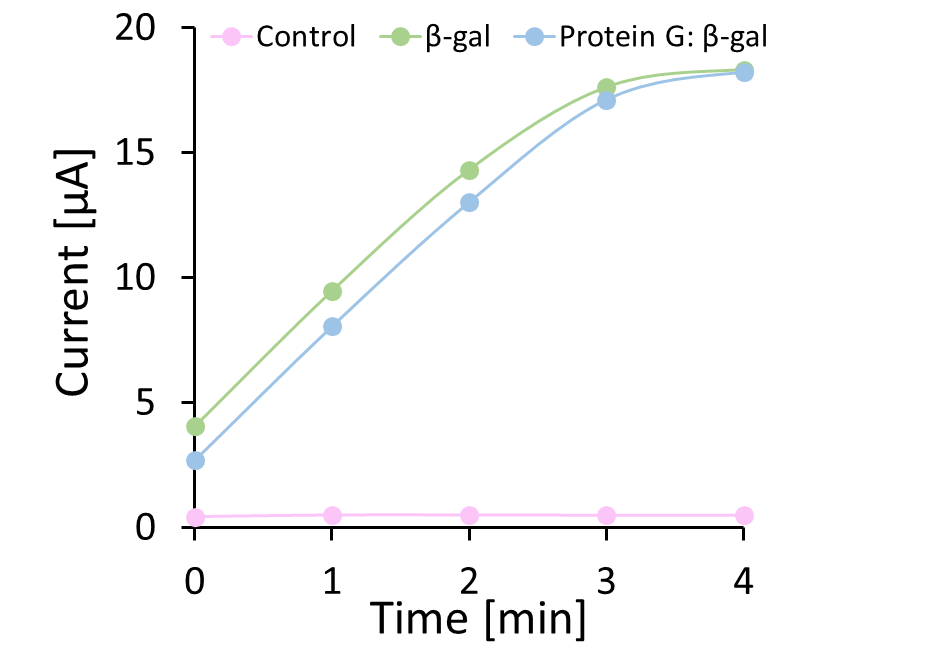

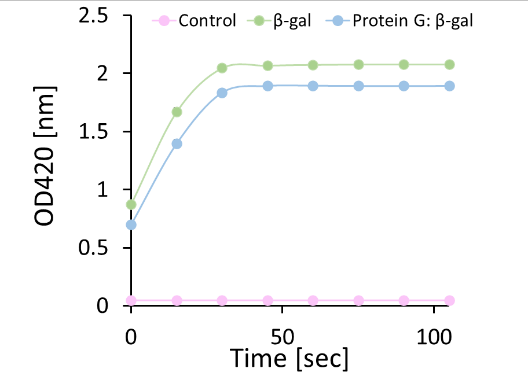


**Figure S3.** (A) The peak oxidative current of the cyclic voltammograms was recorded for the electrochemical studies. The cyclic voltammogram pictured is a measurement of a protein G: β-gal (31.25 μg/mL) and PAPG (0.5 g/L) solution that was taken from -0.1-0.4 V at a scan rate of 0.05 V/s.

(B) The β-gal control was expressed (in *E. coli*) and purified in an identical manner to protein G: β-gal. Protein G: β-gal (1 μM), β-gal only (1 μM), and a buffer control were evaluated spectrophotometrically (left) and electrochemically (right) in an identical manner as in the Methods section. For both spectrophotometric and electrochemical detection, reaction rates for protein G: β-gal (2.27 Abs/min; 5.35 μA/min) and β-gal only (2.34 Abs/min; 5.41 μA/min) were not minimally different. (C) Analysis of freeze thaw stability for protein G: β-gal. For a freeze-thaw cycle, protein G: β-gal (25 μg/mL) samples were frozen at -80°C and subsequently thawed at room temperature. Samples were then measured spectrophotometrically (left) and electrochemically (right) in an identical manner as in the Methods section. Protein G: β-gal maintained its spectrophotometric and electrochemical reaction rates (shown in the table) for two freeze thaw cycles (reaction rates within 10% variance of initial response).


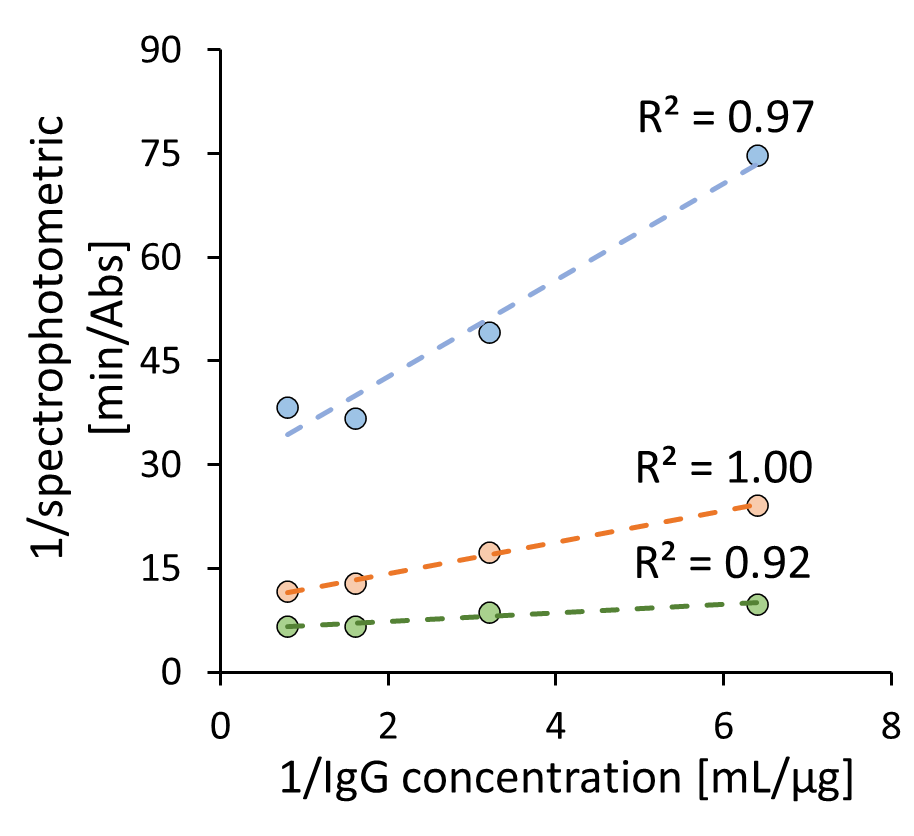


**Figure S4.** Lineweaver-Burke plot of concentration dependent response results from Figure 3 shows linearity of response across protein G: β-gal concentrations: 0.1 g/L (blue), 0.01 g/L (orange), and 0.001 g/L (green).


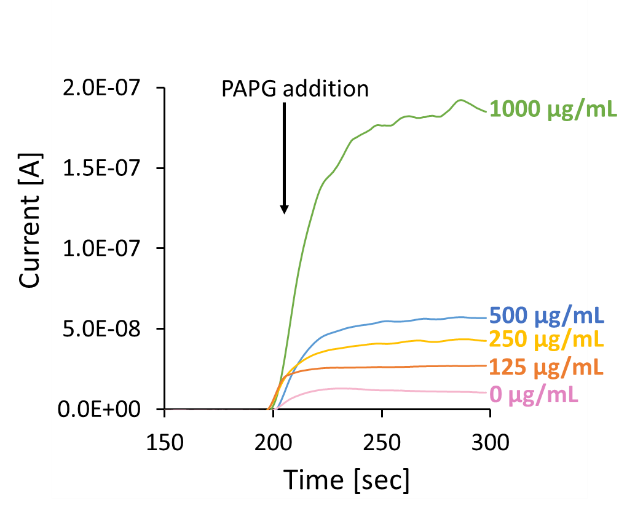


(B)

(C)


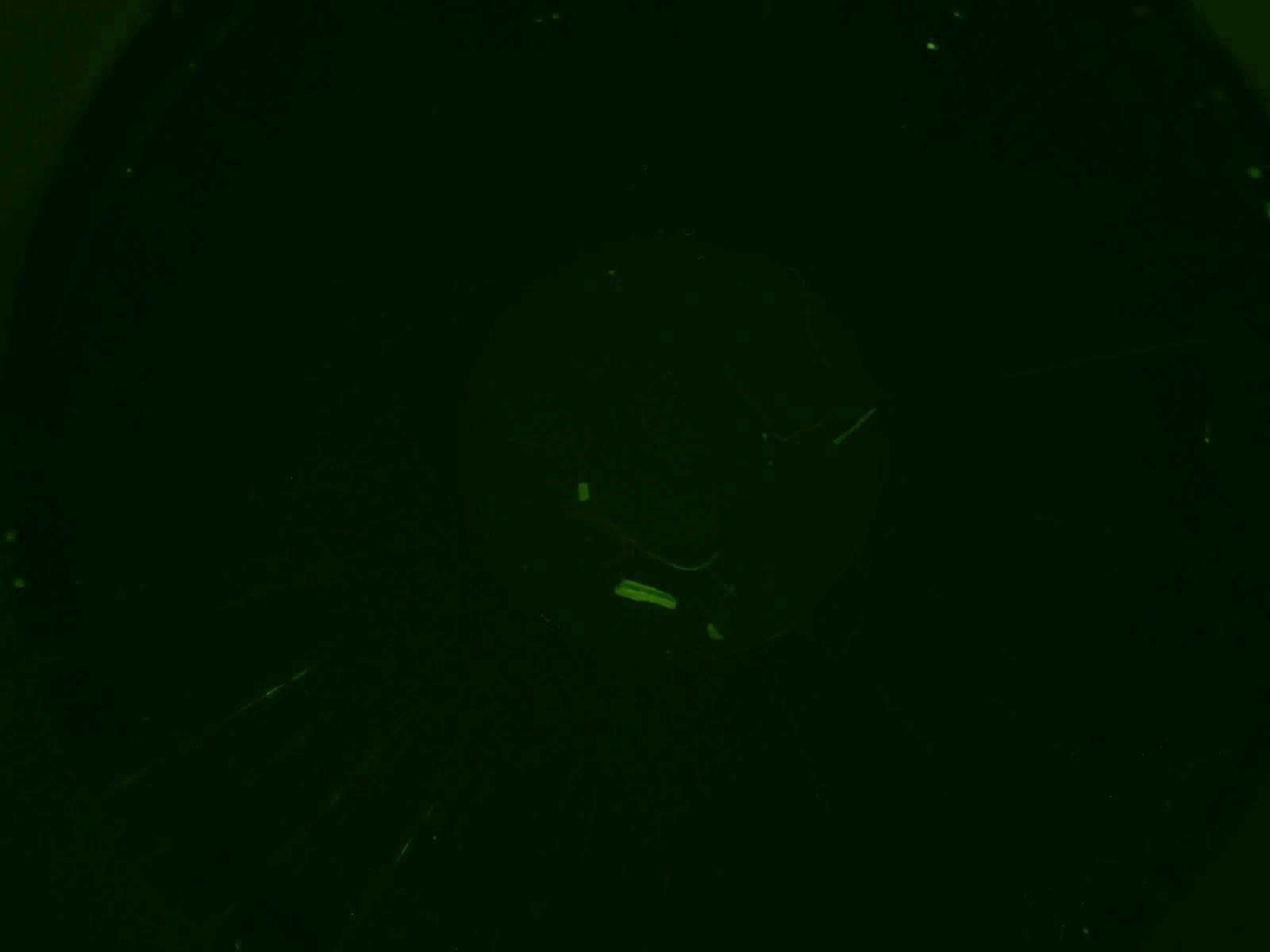


**(i) Without IgG**


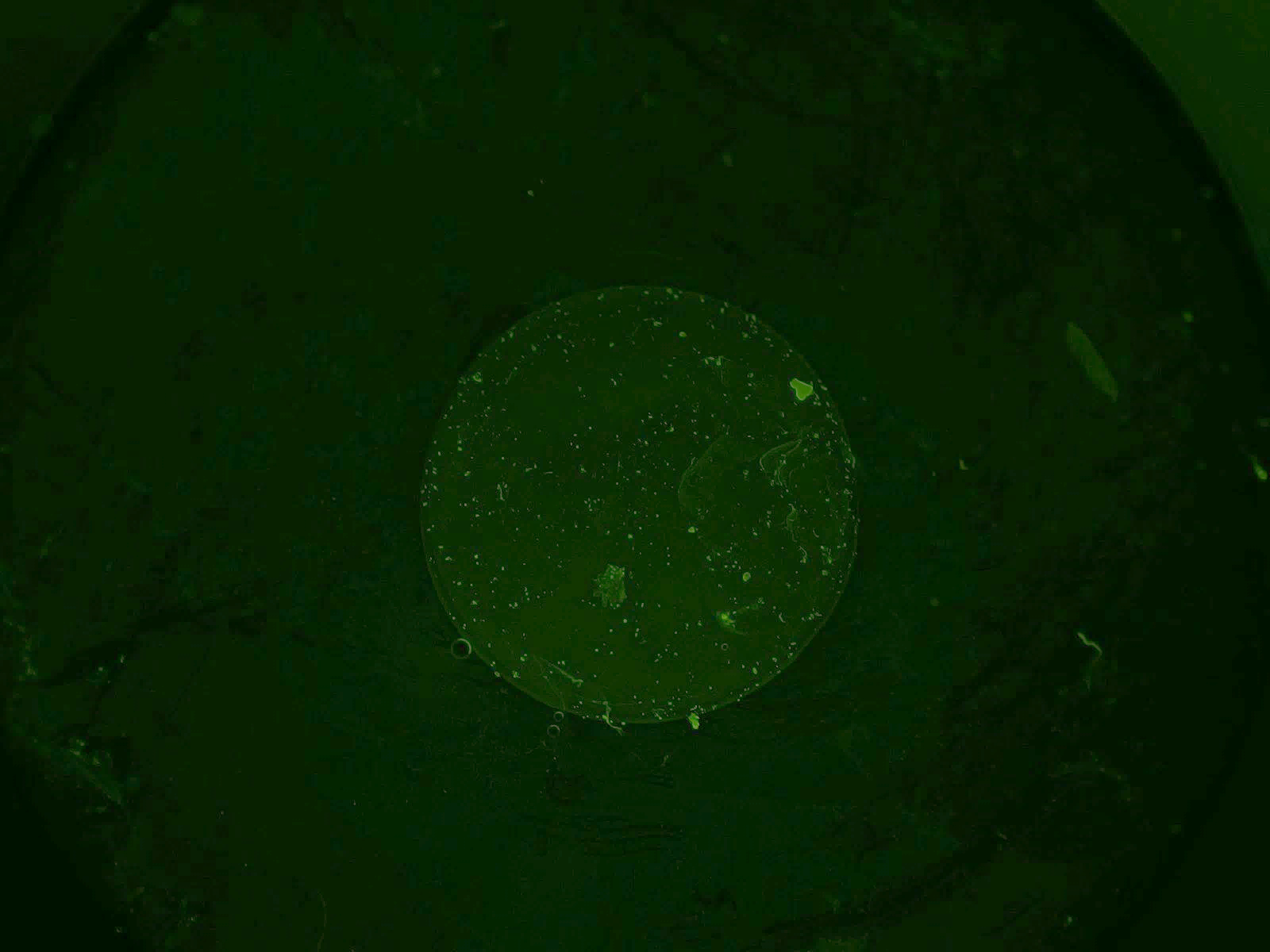


**(ii) With IgG**

(A)


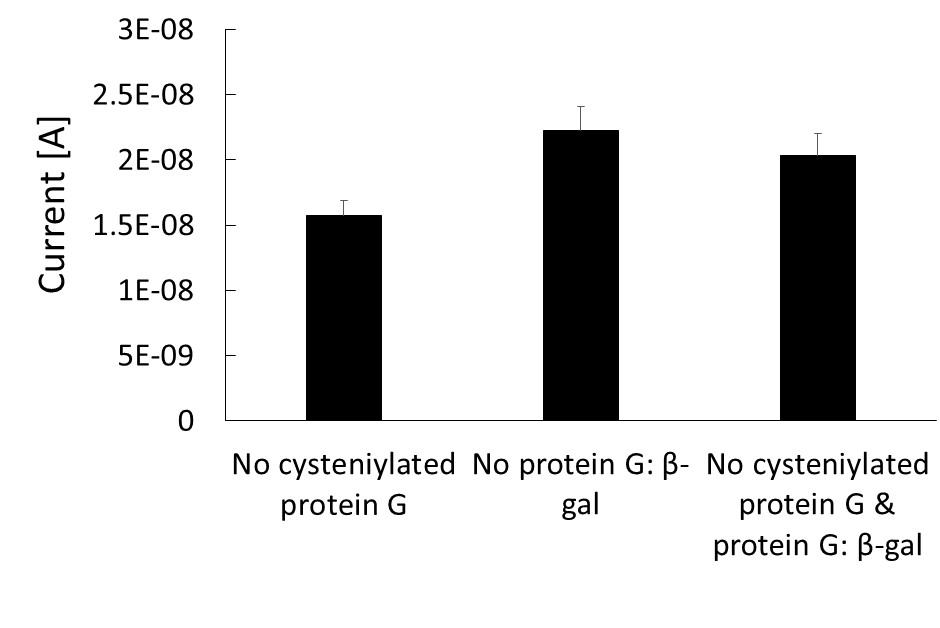

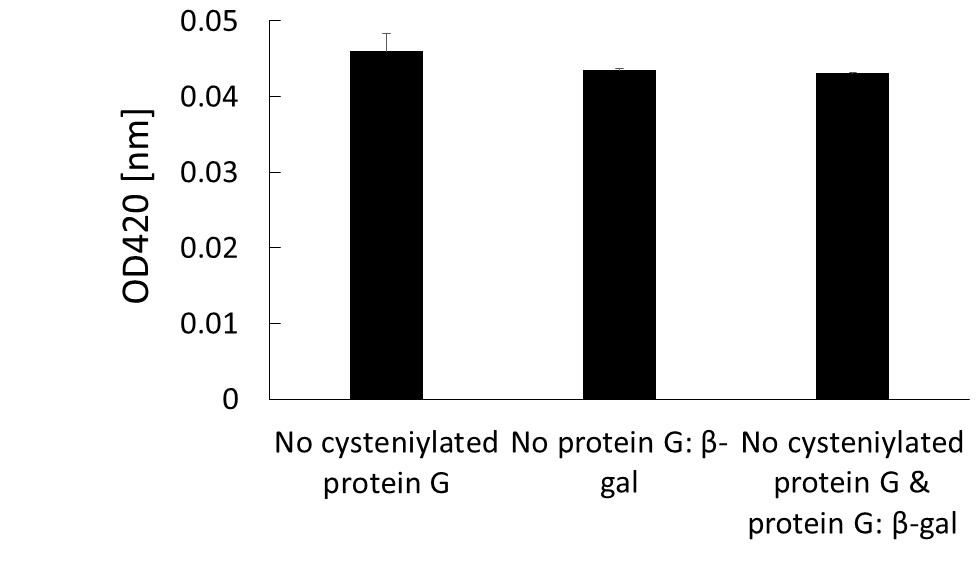


**Figure S5. (**A) FITC-labelled protein G: β-gal was used to show that the fusion protein binds specifically to IgG on the surface of the sensor interface. The interfaces were incubated with: (i) 0.01 protein G: β-gal only or (ii) with IgG (1 g/L) and 0.01 g/L protein G: β-gal. When the interface is not incubated in IgG, a weakly fluorescent signal can be observed. This indicates that the FITC-labelled protein G: β-gal does not bind to the interface surface. However, when the interface is incubated sequentially in IgG and protein G: β-gal, a strong fluorescent signal that is localized on the gold surface of the electrode is clearly visible. These data confirmed that protein G: β-gal is specifically binding to the IgG on the surface of the interface. (B) Representative current responses from the interfaces that were incubated in different IgG concentrations (0-1000 μg/mL) and 0.01 g/L protein G:β-gal. (C) As expected, sensor interface controls for spectrophotometric (left) and electrochemical (right) detection show minimal response.
